# Current Research on Small Circular Molecules: A Comprehensive Overview on SPHINX/BMMF

**DOI:** 10.3390/genes15060678

**Published:** 2024-05-24

**Authors:** Diana Habermann, Charles M. A. P. Franz, Martin Klempt

**Affiliations:** Department of Microbiology and Biotechnology, Max Rubner-Institut, Federal Research Institute for Nutrition and Food, Hermann-Weigmann-Straße 1, 24103 Kiel, Germany; charles.franz@mri.bund.de (C.M.A.P.F.); martin.klempt@mri.bund.de (M.K.)

**Keywords:** SPHINX, BMMF, small circular DNA, cancer, neurodegeneration

## Abstract

Several years of research into the small circular DNA molecules called SPHINX and BMMF (SPHINX/BMMF) have provided information on several areas of research, medicine, microbiology and nutritional science. But there are still open questions that have not yet been addressed. Due to the unclear classification, evolution and sources of SPHINX/BMMF, a risk assessment is currently not possible. However, risk assessment is necessary as SPHINX/BMMF are suspected to be involved in the development of cancer and neurodegenerative diseases. In order to obtain an overview of the current state of research and to identify research gaps, a review of all the publications on this topic to date was carried out. The focus was primarily on the SPHINX/BMMF group 1 and 2 members, which is the topic of most of the research. It was discovered that the SPHINX/BMMF molecules could be integral components of mammalian cells, and are also inherited. However, their involvement in neurodegenerative and carcinogenic diseases is still unclear. Furthermore, they are probably ubiquitous in food and they resemble bacterial plasmids in parts of their DNA and protein (Rep) sequence. In addition, a connection with bacterial viruses is also suspected. Ultimately, it is still unclear whether SPHINX/BMMF have an infectious capacity and what their host or target is.

## 1. Introduction

The existence of small circular DNA molecules in eukaryotic and prokaryotic organisms has been known for decades [1,2]. However, the question of how they got there and what function they serve in some cases still remains unknown. Following the discovery of small circular DNA molecules in tissues and cells associated with neurodegenerative diseases and cancer, research in this field has gained impetus and new insights have been reported. The central question here was whether and how self-replicating circular DNA molecules contribute to the development of cancer or neurodegenerative diseases. Therefore, various theses and research results have been published and will be summarized and evaluated in this review in order to summarize and simplify the assessment and the interpretation of the data published to date. This overview addresses neither circular DNA that is homologous to the chromosomal DNA nor the mitochondrial DNA of the eukaryotic host. Furthermore, this review will consider whether these small circular DNA molecules have structures that are similar to those of bacterial plasmids or viruses.

Long before the discovery of small circular molecules that are focused on here, it had already been postulated that there is a certain factor in the meat of domestic cattle (*Bos taurus*) that appeared to promote the development of cancer [3]. The basis of the reasoning behind this claim was that the excessive consumption of red meat causes an increased risk of bowel cancer [4,5] and, in particular, that meat from domestic cattle is often consumed not fully cooked [3]. For this reason, a previously unknown bovine factor was postulated that appeared to be “clearly heat-stable, replication-incompetent in human cells and possibly structurally related to polyomaviruses” [3]. This hypothesis was underpinned by a further publication in which an attempt was made to demonstrate the dependency of increasing colon carcinoma cases in various countries on the increased consumption of beef [6]. A name was then coined for the then-elusive unknown factor, i.e., bovine meat factor, which was later changed to bovine meat and milk factor (BMMF). In the following years, the postulated BMMFs (bovine meat and milk factors) were found in samples of domestic cattle (serum and milk) as well as in samples of multiple sclerosis patients [7,8,9]. They showed a significant relationship to the previously discovered and published SPHINX molecules, which were isolated from transmissible spongiform encephalopathy (TSE)-infected cells and tissues [10]. Then, in 2019, a press conference held by the German Cancer Research Center (DKFZ) attracted attention, in which the BMMFs were presented as “factors” or “novel pathogens” originating from European domestic cattle (*Bos taurus*) and which were stated to be involved in the development of cancer [11]. As there were considerable doubts regarding the interpretation of existing data by the DKFZ, several further studies were subsequently published by various groups [12,13,14,15,16,17,18,19].

## 2. Epidemiological Considerations

First, a closer look will be taken at the epidemiological data on which the theory of a connection between the consumption of meat and milk (products) from domestic cattle and the development of cancer is based. In the case of intestinal cancer, previous studies have indicated that the consumption of red meat and processed red meat correlates with the incidence of colorectal cancer [20,21,22,23,24]. However, red meat does not only refer to meat of taurine origin but also refers to all meat from warm-blooded animals such as pigs and small ruminants [25]. Further, a high consumption of milk and dairy products was shown to be associated with a reduced risk of colorectal cancer [23,24]. Neither the consumption of red meat nor cow’s milk leads to an increased incidence of breast cancer [24]. Furthermore, an analysis of 21 cohort studies on a total of 1.1 million women shows no connection between milk consumption and the risk of breast cancer [26]. Interestingly, a recently published umbrella review [27], which considered 30,818 cases with 1,378,932 subjects, found no association between the consumption of (cow milk) cheese and increased cancer mortality in general, or with a specific type of cancer. Nor was it possible to demonstrate a cancer-promoting effect of cheese consumption on the incidence of either bowel cancer or breast cancer [27]. In addition, the increased consumption of cheese was shown to significantly reduce the incidence of estrogen-independent breast cancer [27]. In another recent prospective study from the United Kingdom, over 500,000 test subjects were examined with regard to their dietary behavior. The amount of protein consumed from the individual dietary components was calculated and correlated with different types of cancer [28]. Totals of 1193 cases of colorectal cancer and 2024 cases of breast cancer occurred during the observation period (4 years). It was shown, however, that the intake of proteins from dairy products and milk was inversely associated with the occurrence of colorectal cancer [28]. Cheese intake alone does not influence the incidence of colorectal cancer [27]. No association with the intake of proteins from milk or dairy products could also be found for the incidence of breast cancer [28]. In summary, it can be said that no increased risk of colon cancer or breast cancer linked to the intake of milk or dairy products could be established to date. Furthermore, the association between the consumption of red meat and the occurrence of colon carcinomas is not exclusively related to beef [24], so a taurine-specific effect cannot be proven.

## 3. Is SPHINX/BMMF DNA Viral or Plasmid DNA?

In order to investigate whether SPHINX/BMMFs are only present in *B. taurus*-derived food products, further food products and animal samples were examined [13,14,15,16,18,19]. All these studies are based on the methods of isolation and detection of SPHINX/BMMF DNA previously described by the DKFZ, using SPHINX/BMMF-specific primers to amplify these molecules from Round Circle Amplification (RCA)-enriched food DNA samples. Using primers specific for the rep gene present in SPHINX1.76/BMMF group 1, SPHINX2.36/BMMF group 2 or related sequences, König et al. [13] were able to show that these DNA molecules could be detected at a high incidence in milk from water buffalo (83% in one herd and 100% in another herd). Using identical (or similar) primers, circular DNA similar to SPHINX1.76/BMMF group 1 was also detected in sheep and goat milk [14]. Later, in our studies [16], we were able to show that DNA fragments with very high homologies to SPHINX1.76/BMMF group 1 or SPHINX2.23/BMMF group 2 also occurred in 143 various food samples from local markets, including white and red meats, seafood, fruits, vegetables, grain and baby food, as well as in individual animal samples from pork (feces, saliva) and chicken (feces). In our further investigations, we showed that complete circular DNAs with high homologies to SPHINX/BMMF could also be detected in foods from animals and plants, including pork, wild boar, chicken meat, Alaska pollock, pangasius, black tiger shrimp, apple, carrot, and sprouts from alfalfa, radish, and broccoli [19]. Further SPHINX/BMMF molecules had been isolated from blood and feces of African and Asian cattle [18].

Based on the nucleotide sequences of all known SPHINX/BMMF molecules, fundamental structural properties can be summarized. First of all, it should be mentioned that zur Hausen et al. [29] categorized the SPHINX/BMMF molecules known to date into four different groups. Groups one and two contained SPHINX1.76 and SPHINX2.34 isolated by Manuelidis [10], respectively. The third group consisted of the gemycircularvirus-related (*Genomoviridae*) isolates from healthy cattle serum and milk. The MSSI1.162 DNA sequence isolated from the serum of an MS patient and showing homology to a *Psychrobacter* spp. plasmid was classified in the fourth group. In this review, we focus only on the group 1 and 2 SPHINX BMMF members.

**Figure 1:**
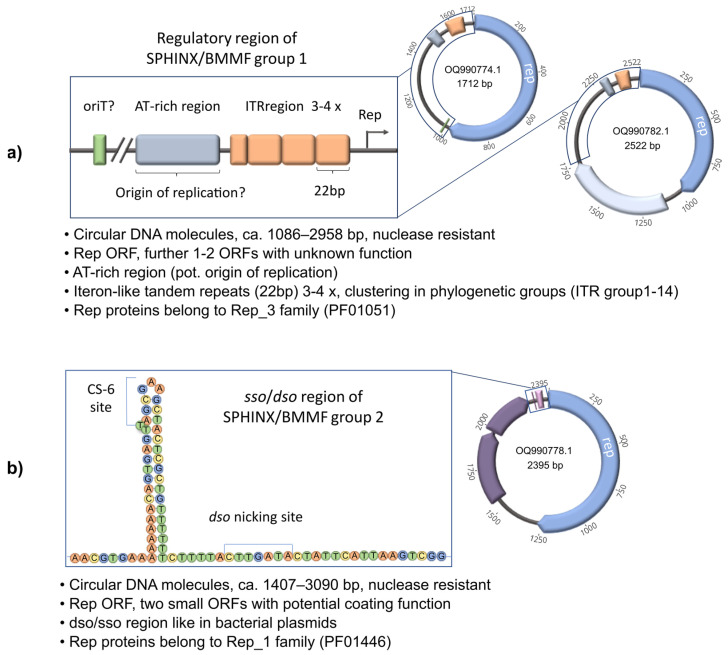
Overview of SPHINX/BMMF group 1 and 2 molecules. (**a**) Summary of the properties of SPHINX/BMMF group 1 molecules known to date and illustration of two typical molecules including one (Rep ORF in blue) or two ORFs (second ORF in light blue) [10,13,14,18,19,30]. Schematic image of the regulatory region (framed), which potentially includes *ori*T (green) [19], AT-rich region (potential origin of replication, grey) [19,31] and iteron-like tandem repeats (ITRs, orange) [19]. (**b**) Summary of the properties of SPHINX/BMMF group 2 molecules known to date and illustration of a typical molecule including one Rep ORF (blue) and two further ORFs (purple) [10,13,18,19,30,32] and a schematic sketch (secondary structure of ssDNA) of the potential *sso*/*dso* region (framed) and the typical *sso* recognition site (CS-6) and the *dso* nicking site [19].

To compare the structure and relationship to previously identified SPHINX/BMMF molecules, comparative sequence analyses as well as phylogenetic analyses have been performed [19]. As mentioned before, Manuelidis showed that the isolated molecules are circular, because they could be enriched using Phi29 Polymerase (rolling circle amplification). The circular SPHINXs have been isolated from TSE infectious material that was treated very intensively with nuclease, so it is assumed that the DNA was protected from digestion by the involvement of proteins [10]. The sizes of the SPHINX/BMMF group 1 representatives (to date) range between 1086 bp and 2958 bp. The sizes of the SPHINX/BMMF group 2 representatives (to date) range between 1407 bp and 3090 bp. To date, 59 isolates of the SPHINX/BMMF group 1 and 127 isolates of the SPHINX/BMMF group 2 are known. All members of the two groups contain a replication initiation protein with high sequence homologies (over 70%) to replication proteins of *Acinetobacter* plasmids [30] or *Acinetobacter* phages [10]. The replication proteins of the SPHINX/BMMF group 1 molecules belong to the Rep_3 family (PF01051), whereas the Rep proteins of SPHINX/BMMF group 2 belong to the Rep_1 family (PF01446). Some of the members of SPHINX/BMMF group 1 have a second ORF encoding a protein with a function that is still unclear, but the involvement of this protein in transcription performance was assumed [31]. The members of SPHINX/BMMF group 2 also contain two small ORFs that also have unknown functions, but in the work of Longkumer et al. [32], a function of the ORF products as envelope proteins was proposed. Interestingly, in that work, similarities between the plasmid pTS236 and the SPHINX/BMMF group 2 member SPHINX2.36 were confirmed. For both, pTS236 and SPHINX2.36 were proposed to be similar at the nucleotide level to the bacterial virus AbDs1. Between the members of the SPHINX/BMMF group 1 and 2, several differences were also found in the non-coding regions. In SPHINX/BMMF group 1 molecules, a 22 bp iteron-like tandem repeat (ITR) directly upstream of the rep ORF with three to four repetitions is highly conserved [10,19,30]. In addition, there is an AT-rich region upstream of the rep ORF and the ITR [19,31]. The interpretations of in silico analyses differ between the publications regarding the motifs located in the AT-rich region. Manuelidis found 10-mer repeats with minor variations and a 3.7× periodicity [10]. De Villiers et al. [30], in turn, suspected a viral origin of replication, because the putative origin of replication was similar to the nonanucleotide stem-loop origin of replication in single-stranded plant and animal viruses [30]. Additionally, Habermann et al. [19] found similar sequences to DnaA boxes. It turned out that a viral origin of replication is very unlikely, because the proposed sequence of 12 bp is too short to form a functional stem-loop motif, which usually consists of an appr. 32 bp sequence and a conserved nonanucleotide sequence ([T/A]A[A/T/G]TTATAC, [33]). Whether there are DnaA boxes or short tandem repeats which act as recognition sequences for replication (origin of replication) has to be further experimentally elucidated. In addition, several of the SPHINX/BMMF group 1 molecules also contain conserved sequence motifs with similarities to *ori*T elements, which are important for the transfer of plasmids from one host to another. Group 2 SPHINX/BMMF molecules, on the other hand, contain a highly conserved *dso* region, which is also found in several plasmids that replicate via rolling circle replication. Further, a *sso* region can be detected, which is more variable than the *dso* region. These clear differences in the non-coding region have led to the assumption that SPHINX/BMMF group 1 and 2 molecules have different replication mechanisms that are more similar to plasmid replication than to virus replication [19]. The features of the SPHINX/BMMF group 1 and 2 molecules mentioned above are summarized in Figure 1. Finally, whether the circular SPHINX/BMMF molecules are single or double stranded has not yet been clearly demonstrated, as both versions were detected in the analyses of Manuelidis [10] using BzNp DEAE chromatography, showing a higher proportion of ssDNA molecules than dsDNA molecules in 263 K scrapie-infected hamster brain preparations. Comprehensive investigations into the single- or double-stranded nature of this DNA have not yet been carried out but are needed to clearly determine whether the SPHINX/BMMF molecules are viral or plasmid elements, which is currently still not clear. However, it is quite conceivable that single-stranded DNA and double-stranded DNA are present together if the SPHINX/BMMFs are plasmids, as proposed by Habermann et al. [19].

**Figure 2:**
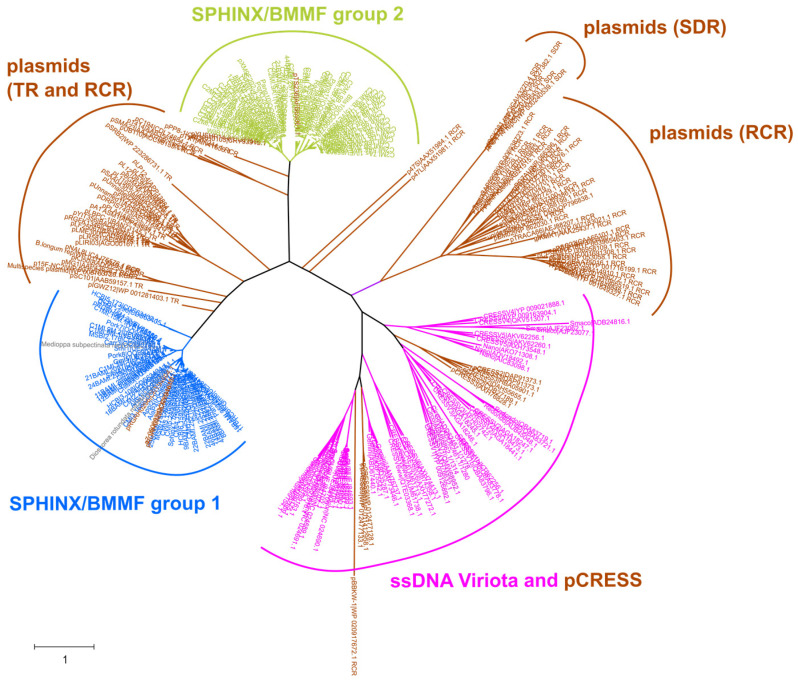
Radiation tree of the phylogenetic analysis of Rep proteins of SPHINX/BMMF group 1 (blue), of SPHINX/BMMF group 2 (green), of plasmids (brown) and also including pCRESS plasmids and ssDNA viruses (pink). The replication mechanisms of rolling circle replication (RCR), theta replication (TR) or strand displacement replication (SDR) of the plasmid cluster are indicated in brackets. The tree was calculated using the maximum likelihood method and Whelan and Goldman model and information on the used sequences is given in the supplemental data of Habermann et al. [19].

Phylogenetic analyses of the isolated SPHINX/BMMF sequences where performed in several studies [13,14,19,30]. The relation to the proposed SPHINX/BMMF groups [30] could be confirmed. Furthermore, the phylogenetic trees of the members of BMMF groups 1 and 2 showed a distinct cluster formation. Whereas the SPHINX/BMMF group 2 tree branched into two big clusters, the SPHINX/BMMF group 1 tree branched into several smaller clusters. Notably, each cluster showed an individual ITR sequence. Therefore, clustering into ITR groups was proposed by Habermann et al. [19]. This grouping was confirmed by an analysis of the truncated Rep protein sequences. These Rep protein sequences contained highly conserved amino acid motifs, which are able to recognize the ITR sequences [19]. In total, 14 individual ITR groups could be identified, of which ITR group 3, which is the focus of several studies concerning their neurogenerative or cancerogenic potential, contains the most members. Finally, a phylogenetic analysis of the Rep protein amino acid sequences of several ssDNA viruses, plasmids (including members of different replication mechanism types) and the then-known SPHINX/BMMF group 1 and 2 members revealed a more significant relationship between the SPHINX/BMMF Rep proteins and the Rep proteins of plasmids than with the Rep proteins of ssDNA viruses (Figure 2).

Specifically, the relation of SPHINX/BMMF group 2 to rolling circle replicating plasmids fits with the detected conserved *dso*/*sso* regions mentioned above, because these *dso*/*sso* regions were also found in rolling circle-type plasmids [19]. The question which then remains is if SPHINX/BMMFs are plasmids, in which host are these contained? High sequence similarities (over 70%) to Acinetobacter plasmids have been found, so could these possibly serve as hosts? This question still remains to be elucidated. On the other hand, there are also indications that SPHINX/BMMF could be bacteriophages. In this case, however, Acinetobacter could also act as their host. Clearly, these are unsolved questions which should be addressed in further studies.

## 4. Involvement of SPHINX/BMMF in Neurodegeneration

Over recent decades, the involvement of viruses in different neurodegenerative illnesses has often been proposed [10,34,35,36]. In the course of research on transmissible spongiform encephalopathies (e.g., Creutzfeldt–Jakob disease, scrapie), two circular DNA sequences of 1.76 and 2.36 kb were isolated from N2a-22L cells (N2a infected with 22L prions) by Manuelidis in 2011 [10]. These DNA sequences, called “SPHINX 1.76 and SPHINX 2.36”, were also detected in brain preparations from 263 K scrapie-infected hamsters and from FU-CJD-infected mice at very low levels in uninfected cells and brain preparations [10]. However, more than 2500-fold concentrations of SPHINX were found in infected cells. In further work by this research group, a polyclonal affinity-purified antibody directed against the 41 kDa Rep protein (here, called Spx1) encoded by SPHINX 1.76 was used to show that the protein was present in neural cells and the brains of different mammals. Interestingly, the Spx1 protein was present in much higher concentrations in synapses than in the perikaryon. It is possible that SPHINX DNA, like mitochondrial DNA, is processed in the synaptic regions. Since a remarkably high concentration of the Spx1 protein was found in synapses, the authors assumed a functional advantage for the neuronal cell in the presence of the Spx1 protein [37]. The Rep protein of SPHINX2.36 (27 kDa ORF) was not investigated more closely, but the earlier works of Botsios and Manuelidis [38] detected a homologous protein in brain preparations using an antiserum against an environmental bacterial virus of *Acinetobacter*.

Using anti-Spx1 antibodies, the distribution of this protein was analyzed in various tissues in rodents and humans [39]. It was also found that the Spx1 protein is predominantly expressed in cells that are dependent on special cell-to-cell communication. However, the ontogenetic lineage (endo-, meso- or exoderm) from which the cell in question originates seemed to be irrelevant. For example, high expression levels were found in the smooth muscles of the uterus, arteries, gastrointestinal tract, pancreatic β cells, etc., whereas no Spx1 could be detected in striated muscles, lung alveoli, hepatocytes and bone. Further investigations showed that Spx1 is mainly found in the dendritic claw of the excitatory granular cells (GCs) of the cerebellum. These cells synchronize the temporal and spatial coordination of mossy fiber cells signals by responding only to the simultaneous activation of multiple mossy fiber cells [40]. GCs may be involved in the control of neuronal oscillation [41]. The investigations of the cerebellums of various animals and humans show that Spx1 may be involved in neurodegenerative processes and could possibly be used as an early marker. Furthermore, several works indirectly confirmed the possible involvement of SPHINX in neurodegenerative diseases, because they isolated four sequences which could be assigned to the SPHINX/BMMF groups 1, 3 and 4 [29] (see chapter 3) from the sera of multiple sclerosis patients [7,8,42]. Moreover, it should be noted that in the work of Eilebrecht et al. [31] the SPHINX/BMMF Rep protein was detected in 20 out of 30 samples of blood sera from healthy patients. This means that there is obviously no direct correlation between the disease and the presence of the SPHINX/BMMF Rep protein in blood sera.

Since the expression of Spx1 decreases during spermatogenesis to such an extent that it cannot be detected in mature sperm, but increases significantly during ovogenesis up to ovulation, maternal inheritance was assumed [39]. Furthermore, the theory of an ancient origin or infection was postulated [39], which should be evaluated in further studies concerning the evolution of these molecules. A causal involvement of the SPHINX/BMMF Rep proteins in neurodegeneration has of yet not been clearly demonstrated. The occurrence of SPHINX/BMMF DNA molecules in cells or tissues and the detection of SPHINX/BMMF Rep proteins are summarized in Table 1.

## 5. Involvement of SPHINX/BMMF in Cancer Development

The initial epidemiological considerations that have been presented in the introduction have led to the isolation of circular DNA from bovine sera [7,8,9,46]. However, these indications gave rise to further investigations into the possible involvement of SPHINX/BMMF Rep proteins in cancer development. In order to detect SPHINX/BMMF Rep proteins in tissue, monoclonal antibodies were developed against the Rep protein of H1MSB.1 (MSBI1.176), which belongs to SPHINX/BMMF group 1, and to which SPHINX1.76 also belongs [43].

Using the anti-Rep antibodies allowed for the detection of SPHINX/BMMF antigens in peritumoral colon cancer tissues, but no SPHINX/BMMF antigens were detected in the colon cancer tumor tissue. The presence of SPHINX//BMMF group 1 antigens was confirmed by Western blot analysis and the isolation of SPHINX/BMMF group 1 DNA using laser microdissection. The co-localization of interstitial macrophages and the Rep protein was observed and immunohistochemically quantified. Significantly increased levels of Rep protein and CD68+ macrophages in cancer tissues (n = 7) compared to cancer-free tissues (n = 8) were observed. Further chronic inflammation markers like reactive oxygen species (ROS) and reactive nitrogen species (RNS) were also detected at elevated levels. Bund et al. assume that this could lead to random DNA mutations of Ki67+ epithelial cells (crypt stem cells) and this could promote their development to progenitors of polyps and colon cancer [43]. Subsequently, these colon cancer tissues and further tissue samples from lung cancer and pancreatic cancer were investigated thoroughly with immuno-electron microscopy (IEM) and fluorescence microscopy. Rep protein detection could be verified for all tissues, especially in the adjacent CD68+ macrophages and in distinct compact particulate pleiomorphic structures [44]. In another study, a clinical cohort of tissues from the healthy colonic or rectal mucosa (n = 10), tumor (n = 26) and tumor-adjacent (n = 29) mucosa of CRC patients was evaluated for Rep and CD68 protein expression (co-immunodetection). In addition, a case-versus-control analysis of a tissue microarray (TMA) of CRC patients (DACHS study, n = 246) using tumor tissues and tumor-adjacent tissues was performed. As before, strong Rep expression in the tumor-adjacent mucosa could be quantified for all the samples. In the tumor tissues and tumor cells, however, the expression was faint or not detectable. Also, the case-versus-control analyses showed differences in the Rep protein expression levels of tumor or healthy cells, so its use as a histological marker for CRC was proposed. The association of Rep expression to clinical–epidemiological parameters was not statistically significant and the authors suggested the inclusion of healthy individuals and parameters like meat and milk consumption as well as generally more tissue sample per patient at variable distances in further studies [45].

A recent publication by Mobaraki et al. [48] describes that in 9 out of 11 patients diagnosed with renal cancer, DNA from SPHINX/BMMF group 1 (5 patients) or 2 (3 patients) or both (one patient) could be detected. In two patients, SPHINX/BMMF group 2 members were detected exclusively in renal tumor tissue; in the other patients, SPHINX/BMMF group 1 or 2 members were detected primarily in peritumoral tissue. In another patient group (n = 152), SPHINX/BMMF group 1 members could be detected in renal tumor tissue in 3.9% of the cases (9 patients) and SPHINX/BMMF group 2 members could be detected in 3.2% of the cases. In only one of these patients, both SPHINX/BMMF group 1 and SPHINX/BMMF group 2 members could be found. In another patient group (n = 39), SPHINX/BMMF group 2 members were shown to be present in nine cases and SPHINX/BMMF group 1 members in one case. Unfortunately, only fragments of the DNA were amplified and the complete SPHINX/BMMF molecules were not presented. Also, the DNA sequences obtained were not deposited in a repository and thus not publicly available. However, from the homologies shown, it could be deduced that very often, the highest homology found was with the sequence of a plasmid of a non-cultivated bacterium. Unfortunately, in this study, it was not stated whether the different SPHINX/BMMF DNA sequences detected in a patient were identical.

To summarize, it can be stated that the postulate that SPHINX/BMMF group 1 members are directly or indirectly involved in the development of cancer is still lacking evidence. In our opinion, the data available to date do not point in this direction, but further investigations are necessary to verify or reject this hypothesis, which is currently still remains a very popular hypothesis. Because of the higher level of rep protein in the peritumoral tissues of CRC and other cancers, Nikitina et al. [45] suggest the detection of SPHINX/BMMF group 1 Rep protein as an early marker, as was proposed for neurodegenerative illnesses [47]. However, it should be mentioned that the antibodies used have not been tested with bacterial or viral Rep proteins in order to test for cross-reactivity. The detection of SPHINX/BMMF DNA molecules in cells or tissues or the detection of SPHINX/BMMF Rep proteins is summarized in Table 1.

## 6. Immunodetection of SPHINX/BMMF Rep Proteins

Since most of the data indicating an involvement of SPHINX/BMMF group 1 members in neurodegenerative diseases or in the development of cancer are based on observations using antibodies as tools, the quality of the used antibodies is of paramount interest. Manuelidis and coworkers [37,39] selected two short amino acid sequences of the SPHINX1.76 Rep Protein (ELDEFRKRIGVLDTEYTR and RNRLSDRFKQGDESA), immunized two rabbits and generated two polyclonal antibodies R1 and R2. Both antibodies reacted in the Western blot with proteins of the expected lengths (41 kDa) and a second band (16 kDa), which has not been identified yet. Through the affinity purification of R1 using the appropriated peptide, the specificity could be significantly increased, so that non-specific bands could not be detected in Western blots using neuronal cells [37]. Bund et al. [43] produced 15 monoclonal antibodies using different approaches. Four antibodies were generated by using two artificially created peptides (EARETGKGINANDPLTVH and KQINEHTDITASYEOHKKGRT). Eleven antibodies were produced using a full-length protein antigen which was affinity-purified prior to use for antigen detection. After intensive testing, three antibodies were used in the following studies: AB3 (epitope mapping = WESKLEEFGVV), AB7 (epitope mapping = QINEHTDITASYE) and AB10 (epitope mapping = WESKLEEFGVV). The antibodies were tested for specificity in several Western blots. In general, an antibody is initially considered specific if only one band is detected in the Western blot [49]. Neither AK3 nor AK10 fulfilled this first criterion: Both show multiple bands in some patient samples. An additional band of around 100 kDa could almost always be detected, which also appeared when the expected band in the 50 kDa range could not be detected. Furthermore, multiple additional bands could be shown to occur in many patient samples, indicating that these antibodies are not specific. Furthermore, both antibodies (AK3 and AK10) led to least three additional bands in the range of 90 to 130 kDa in the positive control, which was the bacterially expressed Rep of a SPHINX/BMMF group1 DNA. In addition, the length of the band identified as specific differed from the control band, which was the recombinant H1MSB.1 (MSBI1.176) Rep protein. This may be due to the fact that the control protein was bacterially expressed and, thus, different post-translational processes might have caused different migration rates. Interestingly, the epitope recognized by AB3 and AB10 (WESKLEEFGVV) was identical or almost identical in many different bacterial or plant Rep proteins (8 March 2024, detected via pBLAST using default parameters: https://blast.ncbi.nlm.nih.gov/), so, it can be assumed that these antibodies would also react with these Rep proteins. Unfortunately, none of the researchers checked for the cross-reactivity of the antibodies using bacterial or viral Rep proteins.

## 7. Infectious Potential of SPHINX/BMMF

Since infection is defined as the invasion and growth of infectious agents such as bacteria and viruses in the body [50], the infection of cells can be defined as the invasion, survival and replication of these in the cell. There are currently very few studies of SPHINX/BMMF in cell cultures, especially of putative invasion, which has not been addressed. The studies by Manuelidis et al. have shown that SPHINX DNA is present in different mammalian cells in varying concentrations [10,37]. This leads to the assumption that the SPHINX DNA can persist in these cells and can therefore probably be replicated in these cells. Further studies on different tissues of mammals including humans confirmed the distinct differential and tissue-specific expression of Spx1 protein [39,47]. In detail, very closely related cells showed a quantitatively different expression pattern of Spx1. For example, the adrenal cortex (glomerulosa) cells that secrete aldosterone showed strong Spx1 expression, while no Spx1 expression was measured in glucocorticoid-secreting (fasciculata) cells. This is surprising because aldosterone-producing cells can be morphologically and functionally transformed into glucocorticoid-producing cells by the administration of the hormone ACTH. This has led to the assumption that the transcription or translation of SPHINX DNA is regulated at the cellular level [39].

In a publication by Eilebrecht et al. [31], a transcription analysis of four different SPHINX/BMMF group 1 isolates, CMI1.252, CMI3.168, MSBI1.176 (H1MSB.1) and MSBI2.176, which were transfected into HEK293TT cells, was analyzed. HEK293TT (short 293TT) cells are characterized by the fact that they are particularly suitable for the replication of single-stranded viruses. 293TT cells are human embryonic kidney cells that contain an integrated copy of the SV40 genome (293T; [51]) and are stably transfected with the T antigen (293TT; [52]). All four constructs tested were transcribed to a different extent in the 293TT cells, with the effectiveness of transcription varying greatly between the different constructs. CMI3.168 and MSBI1.176 showed the highest transcription rate, with an average of 700 and 927 rpm (read per million of total read pairs), whereas CMI1.252 and MSBI2.176 exhibited the intermediate (average 65 rpm) or lowest level (1 rpm), respectively. So far, there is no clear explanation for this, apart from the fact that the isolates differ in their ITR group. MSBI2.176 is a member of the ITR group 7, whereas CMI3.168, MSBI1.176 and CMI1.252 belong to ITR group 3. However, it is interesting to note that the transcription rate of the SPHINX/BMMF group 1 isolate MSBI 1.76 (H1MSB.1) increases significantly when the SPHINX/BMMF isolates MSBI1.76 (H1MSB.1) and CMI1.252 DNA molecules were co-transfected. Presumably, the second protein encoded in CMI1.252 with an as-yet unknown function influences the transcription rate, but this has not been clarified [31]. This protein contains two motifs with high probability [19]. One motif belongs to the DUF6290 family (PF19807) with a, presumably, DNA binding motif and the other motif, an EVE domain, belongs to the PUA superfamily (PF01878) and could be involved in the regulation of the expression of other genes. As CMI1.252 replicates in the HEK293TT cells over a 14-day timespan, while MSBI 1.76 only does so when it is co-transfected with CMI1.252, it is speculated that the second protein encoded by CMI1.252 is responsible for this [31]. This theory also needs to be tested in more detail. Furthermore, both constructs (CMI1.252 and MSBIB1.76) led to an essentially parallel change in the expression of endogenous RNAs of the transfected 293TT cells. At present, it is still unclear which metabolic pathway in particular is addressed and whether the observed changes in transcription led to a change in the physiology of the cells, but several of the up- or downregulated genes were known to be involved in cancer etiology. In summary, it has been shown that SPHINX/BMMF group 1 molecules can survive and replicate after transfection in human cells (HEK293TT), which have been shown to readily transcribe and replicate small single-stranded DNA viruses [53]. Replication in other cells or the natural invasion of the molecule into mammalian or bacterial cells has not been demonstrated yet.

## 8. Future Perspectives

**Figure 3:**
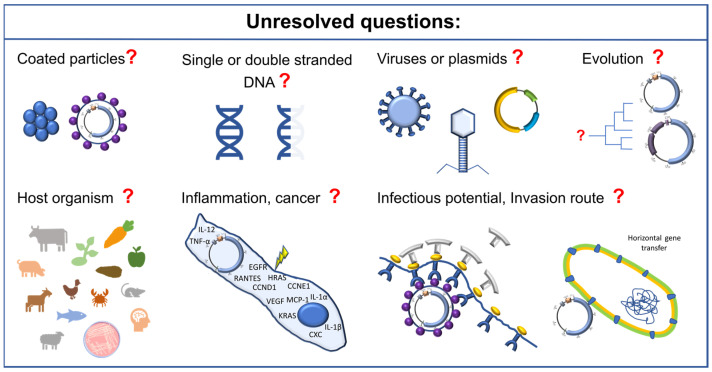
Schematic representation of further important investigations to elucidate the biology and function of SPHINX/BMMF.

As indicated above, there are still numerous open questions relating to the field of SPHINX/BMMF research that need answering, which are summarized in Figure 3. Firstly, it would be highly interesting to investigate the exact biology of these molecules. Are these molecules protected by proteins (which is likely)? If so, what proteins are these? Where are these particles produced? Are SPHINX/BMMF basically single-stranded or double-stranded by nature, or can they be present in both forms, for example, during replication? It would also be important to assess whether these molecules are very ancient in evolutionary terms, as they may have arisen very early in evolution. This would possibly also provide insights into whether these molecules are viral or bacterial in origin. It would also be essential to find out whether a de novo infection of eukaryotic or prokaryotic cells is possible. What would the underlying mechanism of such potential infections be? Would it be possible to prevent an infection? Finally, the physiological influence of SPHINX/BMMF when present within cells would be of particular interest. Under what conditions do SPHINX/BMMF multiply? What is their physiological function? Are there differences between their expression in children and adults? Do they possibly support uncontrolled growth or are they replicated as a reaction of the body to uncontrolled growth and therefore are a part of a defense strategy (Figure 3)? These numerous questions would require quite extensive research to shed more light on these intriguing molecules.

## 9. Conclusions

Previous results showed, that on the one hand, SPHINX/BMMF molecules appear to be an integral component of mammalian cells that are passed on maternally. They are involved in neuronal processes and their link to neurodegenerative diseases is possible but has not yet been clearly demonstrated. A similar picture emerges from the investigations of SPHINX/BMMF in tissues of various types of cancer, where SPHINX/BMMF Rep proteins are also detectable in healthy tissues, albeit in lower quantities than in the vicinity of cancer tissue. On the other hand, due to the diverse (food) sources in which the SPHINX/BMMF molecules were found, it can be assumed that they are ubiquitously distributed. This does not contradict the thesis that SPHINX/BMMF are ancient bacterial viruses that infected mammal cells, but refutes the thesis of an exclusively bovine origin. Comparative analyses of the sequence structures and Rep protein sequences of known SPHINX/BMMF molecules with ssDNA viruses and plasmid sequences indicate higher similarities to plasmids than to ssDNA viruses, but bacterial viruses were not included in this study [19]. It is therefore necessary to investigate whether there are bacterial hosts for SPHINX/BMMF, regardless of whether they are plasmids or bacteriophages. Although the SPHINX/BMMF molecules have been known of for almost two decades, it has not yet been possible to answer all questions about their biology, origin and hazards. It is therefore necessary to further investigate the infectivity of the natural SPHINX/BMMF molecules and their influence on inflammatory processes in order to definitively rule out their involvement in cancer-promoting or neurodegenerative effects.

## Figures and Tables

**Table 1:** Summary of the occurrence of SPHINX/BMMF DNA molecules known to date as well as the detection sites of Rep proteins of SPHINX/BMMF group 1 detected with anti-Rep antibodies.

Occurrence of Circular SPHINX/BMMF DNA Molecules	References
Mouse and hamster cell lines and tissues (TSE-infected)	[10]
Human sera, brain (MS patients), peritumoral colon cancer tissue	[7,8,42,43,44,45]
Bovine milk and blood	[7,9,46]
Water buffalo milk, sheep and goat milk, blood and feces of African and Asian cattle	[13,14,18]
PCR detection in white and red meats, seafood, fruits, vegetables, grain and baby food, feces (pig, chicken) and saliva (pig)	[15,16]
Pork, wild boar meat, chicken meat, Alaska pollock, pangasius, black tiger shrimp, apple, carrot, sprouts	[19]
**Detection of Rep protein ^1^**	**References**
Rodent and human neuronal cells and brain, esp. synapses	[37,47]
Rodent and human smooth muscles of uterus, arteries, gastrointestinal tract, ovary, testis, skin, etc.	[39]
Human peritumoral tissues of colon, lung and pancreas cancer, co-localization with interstitial macrophages, but not in cancer tissue	[43,44,45]

^1^ Antibodies against SPHINX/BMMF group 1 Rep protein.

## Data Availability

No new data were created or analyzed in this study. Data sharing is not applicable to this article.
